# Multi-Protective Effects of Petunidin-3-O-(*trans-p*-coumaroylrutinoside)-5-O-glucoside on D-Gal-Induced Aging Mice

**DOI:** 10.3390/ijms252011014

**Published:** 2024-10-13

**Authors:** Ruinan Wang, Lichengcheng Ren, Yue Wang, Na Hu, Fangfang Tie, Qi Dong, Honglun Wang

**Affiliations:** 1 Qinghai Provincial Key Laboratory of Tibetan Medicine Research and CAS Key Laboratory of Tibetan Medicine Research, Northwest Institute of Plateau Biology, Xining 810008, China; wangruinan@nwipb.cas.cn (R.W.); xklknb@163.com (L.R.); 15353120554@163.com (Y.W.); huna@nwipb.cas.cn (N.H.); fftie@nwipb.cas.cn (F.T.); qdong@nwipb.cas.cn (Q.D.); 2 University of Chinese Academy of Sciences, Beijing 100049, China

**Keywords:** PtCG, D-gal, anti-aging, neuroinflammation, oxidative stress

## Abstract

Petunidin-3-O-(*trans-p*-coumaroylrutinoside)-5-O-glucoside (PtCG), the primary anthocyanin ingredient in *Lycium ruthenicum* Murr., possesses a range of biological activities, including antioxidative properties and melanin inhibition. This study aimed to investigate the protective effect of PtCG on D-galactose (D-gal)-induced aging in female mice and elucidate the underlying molecular pathways. Behavioral experiments, including the MWW and Y-maze tests, revealed that PtCG significantly ameliorated cognitive decline and enhanced learning and memory abilities in aging mice. Regarding biochemical indicators, PtCG considerably improved superoxide dismutase (SOD) and glutathione (GSH) activity while reducing malondialdehyde (MDA) and acetylcholinesterase (AChE) levels in the hippocampus and serum. Furthermore, PtCG ingestion alleviated liver injury by decreasing alanine transaminase (ALT), aspartate transaminase (AST), and alkaline phosphatase (AKP) levels, and attenuated renal damage by reducing blood urea nitrogen (BUN) and uric acid (UA) levels. Transmission electron microscopy (TEM) results demonstrated that PtCG restored the function and quantity of synapses in the hippocampus. Hematoxylin and eosin (H&E), Masson’s trichrome, and Nissl staining revealed that PtCG significantly improved the relevant pathological characteristics of liver and hippocampal tissues in aging mice. The molecular mechanism investigation showed that PtCG downregulated the protein expression of microglial marker ionized calcium-binding adapter molecule 1 (Iba1), astrocytic marker glial fibrillary acidic protein (GFAP), β-secretase 1 (BACE-1), and amyloid-beta_1–42_ (Aβ_1–42_) in the hippocampus of aging mice. The protein expression of inflammatory pathway components, including nuclear factor-kappa B (NF-κB), cyclooxygenase-2 (COX2), inducible nitric oxide synthase (iNOS), and interleukin-1 beta (IL-1β), was also suppressed. These findings suggest that PtCG may possess anti-aging properties, with its mechanism of action potentially linked to the attenuation of neuroinflammation, oxidative stress, and liver and kidney damage. PtCG may have future applications as a functional food for the treatment of aging-related disorders.

## 1. Introduction

Aging is a natural physiological process characterized by material and morphological changes, as well as the functional deterioration of cells, tissues, and organs over time [1,2]. Numerous age-related illnesses, including cancer, diabetes, cardiovascular disease, and neurological disorders, are exacerbated by the aging process. The most notable indicators of aging are cognitive decline, brain atrophy, and substantial hippocampal atrophy combined with brain dysfunction. Although aging is an irreversible process, it can be slowed through various measures such as dietary therapies, herbal interventions, and exercise regimens.

D-gal, a naturally occurring reducing sugar found in the body, can be completely metabolized at lower concentrations and converted to aldose, hydrogen peroxide, and galactose [3,4]. Oxidase, at higher concentrations, catalyzes the transformation of chemicals, resulting in an increased formation of superoxide anion and oxygen-free radical particles. These reactive species can damage macromolecules and impair cellular function [5], ultimately leading to inflammation and immunosenescence. Moreover, the chronic administration of D-gal significantly elevates AChE activity in mice [6]. A substantial body of experimental and clinical evidence supports the notion that prolonged D-gal treatment accelerates the aging process [7]. The oxidative damage, accumulation of metabolites, and cognitive decline observed following D-gal infusion are consistent with the natural aging process [8,9]. Consequently, D-gal has been widely employed in the establishment of animal models for the study of aging and organ damage [10,11,12].

Natural products and dietary supplements have emerged as promising solutions for treating neurodegenerative diseases and age-related dementia due to their antioxidant and anti-inflammatory properties [13]. *Lycium ruthenicum* Murr. anthocyanin has demonstrated significant antioxidant [14], anti-inflammatory [15,16], and anti-tumor effects [17]. In rat studies, it has improved learning and memory deficits, inhibited neuronal apoptosis, and alleviated symptoms by reducing oxidative stress and inflammation while increasing microglial cell viability [18]. PtCG is the primary component of *Lycium ruthenicum* Murr., accounting for over 80% of the total anthocyanin content. Currently, most research focuses on the effects of anthocyanin extracts, with limited studies on the specific anthocyanin monomers, making it challenging to elucidate the material basis and precise mechanisms of action.

To establish the murine aging model, D-gal was administered intraperitoneally. The anti-aging effects were investigated through behavioral tests, biochemical indicator measurements, and histopathological observations of brain, serum, liver, and kidney tissues. The potential mechanism of action was explored by examining the expression of related proteins. This study aims to provide novel insights for the development of innovative anti-aging medications and functional foods targeting age-related diseases.

## 2. Results

### 2.1. PtCG Ameliorated Cognitive Deficits in Aging Mice

**Figure 1:**
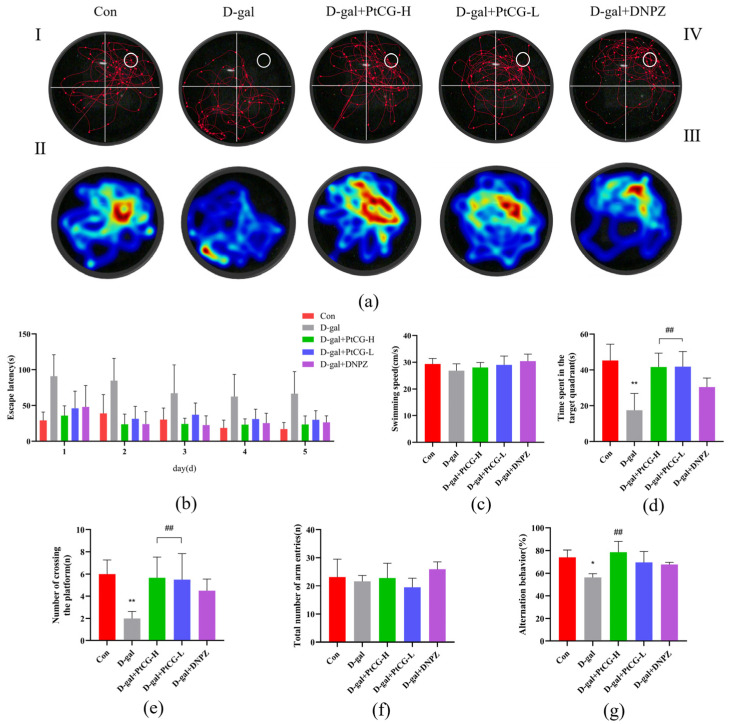
PtCG ameliorated cognitive dysfunction during the behavioral testing of aging mice: (**a**) total swimming paths of the respective groups on the sixth day. Heat map analysis of animal tracking following MWM test. Different colors indicate location preference. Red represents increased time spent and blue represents minimal time spent during trial. I–IV represent quadrant 1–quadrant 4, respectively. The quadrant in which the target platform is located is the target quadrant (quadrant 4). (**b**) The escape latency for five consecutive daily tests. (**c**) Swimming speed in the probe trial. (**d**) Time spent in the target quadrant of the probe trial. (**e**) The number of times mice swam across the target platform in the probe trial. (**f**) The total number of arm entries. (**g**) Percentage alternation in the Y-maze test. Data are expressed as mean ± SD (n = 8 per group). * *p* < 0.05 and ** *p* < 0.01 vs. Con group; ^##^ *p* < 0.01 vs. D-gal group.

As shown in Figure 1, the Morris water maze (MWM) tests demonstrated that PtCG could improve the cognitive deficit of aging mice. Representative trajectories for the different groups are exhibited in Figure 1a. The control group mainly focused on searching for platforms in the target quadrant, whereas mice of the D-gal group searched for target platforms without direction. The mice of the low-dose PtCG (PtCG-L) group all performed better than those of D-gal group but slightly worse than the DNPZ group in the searching mode. In addition, the mice of the high-dose PtCG (PtCG-H) group performed similarly to the control group. During the training period, the latency to find the hidden platform of all mice was shortened. However, the mice of the D-gal group spent more time searching for platforms than those of the control group. Compared with the model group, the latency of mice for the PtCG-L and PtCG-H groups was shortened (Figure 1b). On the sixth day of the trial, the platform was removed to allow the mice to swim freely in order to estimate spatial working memory. The average swimming speed was comparable across all groups, indicating that D-gal did not harm the motor regions of the mice’s brains (*p* > 0.05, F (4, 25) = 1.696) (Figure 1c). However, compared to the control group, the mice of D-gal group swam across the plate fewer times and spent less time in the target quadrant (*p* < 0.01). PtCG treatment could improve the above situation by reducing the amount of time spent in the target quadrant (Figure 1d) (*p* < 0.01, F (4, 25) = 12.02). Nevertheless, PtCG did not have a dose-dependent improvement impact. Although the effect of DNPZ was slightly worse than that of PtCG, it could still significantly improve the cognitive ability of mice (Figure 1e) (*p* < 0.01, F (4, 25) = 6.581). The Y-maze test was another tool used to evaluate cognitive ability. Spontaneous alternation was extensively employed to measure the spatial memory of animals. The D-gal group possessed a lower percentage of alternation than the control group (Figure 1f,g) (*p* < 0.05, F (4, 25) = 1.899, F (4, 15) = 5.834). PtCG treatment considerably increased the proportion of alternation (*p* < 0.01), and it outperformed the DNPZ group.

### 2.2. PtCG Regulated MDA Content and SOD, GSH, AChE Activity of Aging Mice

**Figure 2:**
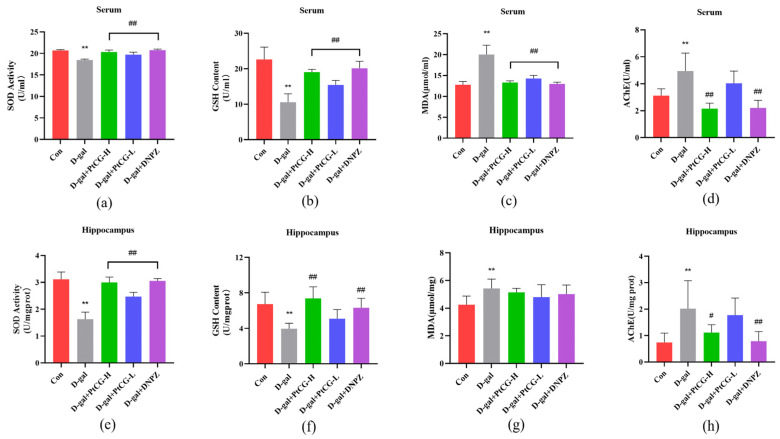
PtCG regulated the MDA content and SOD, GSH, AChE activity in the serum and hippocampus of aging mice. (**a**) Activity of SOD in the serum. (**b**) Activity of GSH in the serum. (**c**) Content of MDA in the serum. (**d**) Activity of AChE in the serum. (**e**) Activity of SOD in the hippocampus. (**f**) Activity of GSH in the hippocampus. (**g**) Content of MDA in the hippocampus. (**h**) Activity of AChE in the hippocampus. Data are expressed as mean ± SD (n = 8 per group). ** *p* < 0.01 vs. Con group; ^#^ *p* < 0.05 and ^##^ *p* < 0.01 vs. D-gal group.

Reduced SOD activity indicates that the antioxidant capacity of nerve cells in patients with Alzheimer’s disease is weakened, leading to an increase in free radicals and neuronal apoptosis [19,20,21]. The D-gal group exhibited significantly lower SOD activity in the serum and hippocampus compared to the control group (*p* < 0.01), while the PtCG and DNPZ treatment groups demonstrated significantly higher SOD activity than the D-gal group (Figure 2a,e) (*p* < 0.01, F (4, 35) = 53.28, F (4, 25) = 54.38). GSH, a glutathione of glutamate, L-Cysteine and glycine, is present in nearly every cell of the body. GSH is crucial for maintaining normal immune function and exhibits antioxidant and integrative detoxification effects [22]. The GSH activity in the hippocampal and serum of the D-gal group was significantly lower than that of the control group, whereas the GSH activity in the PtCG and DNPZ groups was significantly higher than that of the model group (*p* < 0.01, F (4, 35) = 37.16, F (4, 35) = 12.25) (Figure 2b,f). Elevated levels of MDA accelerate the aging process [23]. The MDA levels of the serum and hippocampal of the model group were considerably higher than those of the control group, while PtCG and DNPZ administration significantly reduced MDA levels (*p* < 0.01, F (4, 35) = 57.01, F (4, 35) = 3.614) (Figure 2c,g). Acetylcholine is a classical neurotransmitter, and the biosynthesis and catabolism of acetylcholine are regulated by key enzymes in brain tissue. Therefore, measuring the enzyme activity of AChE in brain tissue can indirectly assess the neurological function of the brain [24]. The model group had considerably higher levels of AChE in the serum and hippocampus compared to the control group. However, PtCG-H and DNPZ therapy significantly reduced these levels (*p* < 0.01, F (4, 45) = 21.98, F (4, 35) = 7.247) (Figure 2d,h).

### 2.3. PtCG Attenuated the Levels of AKP, ALT, and AST in the Liver and the Levels BUN and UA in the Kidney of Aging Mice

**Figure 3:**
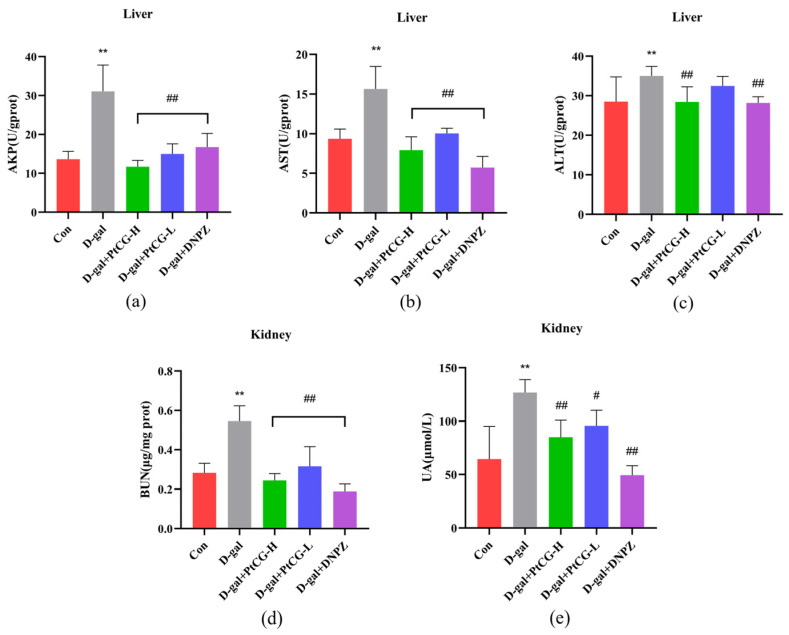
PtCG attenuated AKP, ALT, and AST levels in the liver and BUN and UA levels in the kidney of aging mice. (**a**) Levels of AKP in the liver; (**b**) levels of AST in the liver; (**c**) levels of ALT in the liver; (**d**) levels of BUN in the kidney; (**e**) levels of UA in the kidney. Data are expressed as mean ± SD (n = 8 per group). ** *p* < 0.01 vs. Con group; ^#^ *p* < 0.05 and ^##^ *p* < 0.01 vs. D-gal group.

Long-term D-gal injections in animals induce systemic metabolic abnormalities, leading to organ deterioration that mimics the natural aging process [25,26]. D-gal treatment can cause liver and kidney damage and dysfunction [27]. Compared to the control group, the model group exhibited significantly elevated levels of AKP, ALT, and AST in mice liver (*p* < 0.01, F (4, 45) = 42.26, F (4, 45) = 46.67, F (4, 45) = 7.064). The PtCG-H and DNPZ groups showed significantly reduced levels of these enzymes (*p* < 0.01), with PtCG demonstrating a slightly superior therapeutic effect compared to DNPZ (Figure 3a–c). The D-gal group also had significantly increased BUN and UA levels in the kidneys compared to the control group (*p* < 0.01). Mice treated with PtCG and DNPZ exhibited lower BUN and UA levels compared to the D-gal group, suggesting that both PtCG and DNPZ exerted protective effects against D-gal-induced kidney injury (Figure 3d,e) (*p* < 0.05, *p* < 0.01, F (4, 45) = 44.98, F (4, 35) = 21.98).

### 2.4. PtCG Attenuated Neuronal Damage in the Hippocampus of Aging Mice

**Figure 4:**
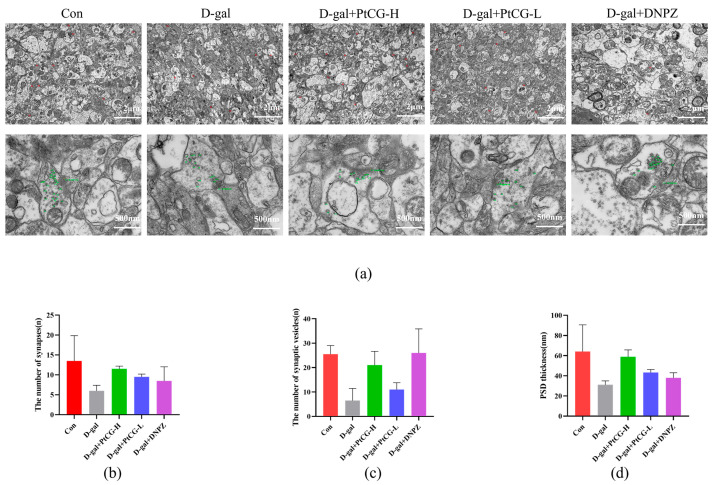

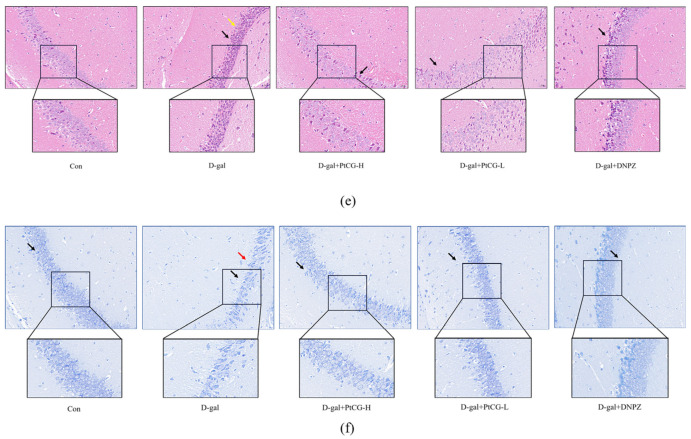
PtCG attenuated neuronal damage in the hippocampus of aging mice. (**a**) Microstructure observation of hippocampus with TEM. Scale bar, 2 μm and 500 nm. The red letter represent each synapse. The green letters and numbers represent each synaptic vesicle and the thickness of PSD. (**b**) The number of synapses; (**c**) the number of synaptic vesicles; (**d**) the thickness of postsynaptic density; (**e**) the hippocampal of mice were observed using H&E staining: magnification 40× and 80×, Neurons shrink (black arrow), neurons become denatured (yellow arrow); (**f**) the hippocampal of mice were observed using Nissl staining: magnification 40× and 80×. The cells atrophied with vacuoles (black arrows) and intercellular spaces (red Arrows). Data are expressed as mean ± SD (n = 3 per group).

To investigate the impact of PtCG on neuronal damage, the hippocampus of aging mice was stained with H&E and Nissl staining. The TEM results revealed that the number of synaptic vesicles and synapses in the hippocampus decreased, and the postsynaptic density (PSD) became thinner following D-gal injection. Moreover, treatment with PtCG-H increased the number of synapses and synaptic vesicles, as well as PSD thickness, compared to the D-gal group (F (4, 10) = 4.482, F (4, 10) = 6.170, F (4, 5) = 2.445) (Figure 4a–d).

H&E staining revealed significant neuronal damage in the hippocampal regions of D-gal-induced mice compared to the control group. The majority of neurons in the D-gal group had shrunk, were deeply stained, and lacked peripheral staining. While the PtCG-L group exhibited persistent neuronal damage, the DNPZ and PtCG-H groups showed improvement in this phenomenon (Figure 4e). Nissl staining results indicated a clear reduction in the number of hippocampal neurons and Nissl bodies in the model group compared to the normal group. Additionally, the model group displayed increased intercellular space, disorganized neuronal arrangement, and a significant decrease in the number of layers, suggesting a partial damage to neuronal morphology and structure following D-gal induction. However, treatment with PtCG and DNPZ increased the number of Nissl bodies and neurons, and the plump cell bodies suggested potential neuroprotective and restorative effects on the damaged neurons (Figure 4f).

### 2.5. PtCG Attenuates Pathological Damage in the Liver of Aging Mice

**Figure 5:**
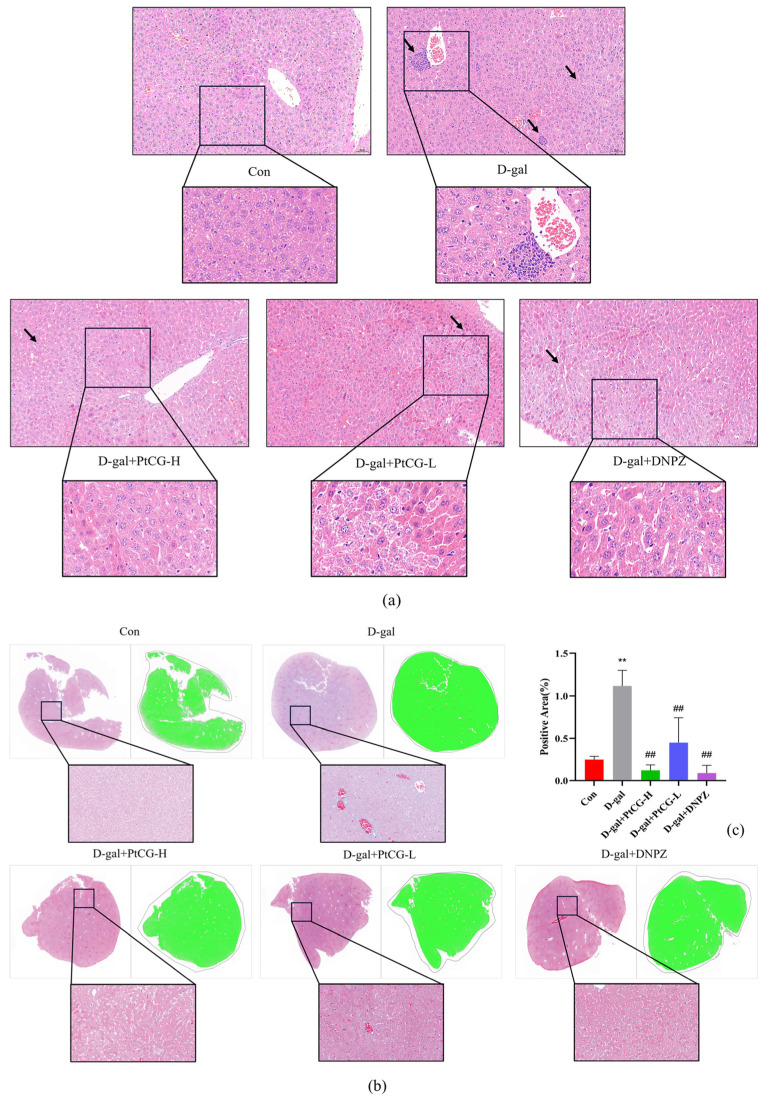
PtCG attenuated pathological damage in the liver of aging mice. (**a**) H&E staining of liver: magnification 40× and 80×. (**b**) Masson staining of liver: magnification 20× and 40×. (**c**) Statistical analysis of the positive areas by Masson staining. Data are expressed as mean ± SD (n = 3 per group). ** *p* < 0.01 vs. Con group; ^##^ *p* < 0.01 vs. D-gal group.

Histological examination via H&E staining revealed substantial hepatocellular damage in D-gal-treated mice, characterized by cytoplasmic vacuolization (black arrowheads), cellular edema, reduced cytoplasmic staining intensity, hepatocyte degeneration, and inflammatory cell infiltration within the sinusoidal spaces. Treatment with PtCG and DNPZ partially restored and mitigated these histopathological alterations (Figure 5a). The quantitative analysis of Masson’s trichrome staining demonstrated a significantly higher positive staining rate in the D-gal model group compared to the control (*p* < 0.01, F (4, 10) = 20.20). The administration of PtCG induced a dose-dependent reduction in the positive rate (*p* < 0.01), comparable to the effects observed with the positive control drug, DNPZ (*p* < 0.01). Collectively, these findings suggest that PtCG and DNPZ exert protective effects against D-gal-induced liver injury in this murine model of aging (Figure 5b,c).

### 2.6. PtCG Decreased the Expression of Iba1 and GFAP in the Hippocampus of Aging Mice

**Figure 6:**
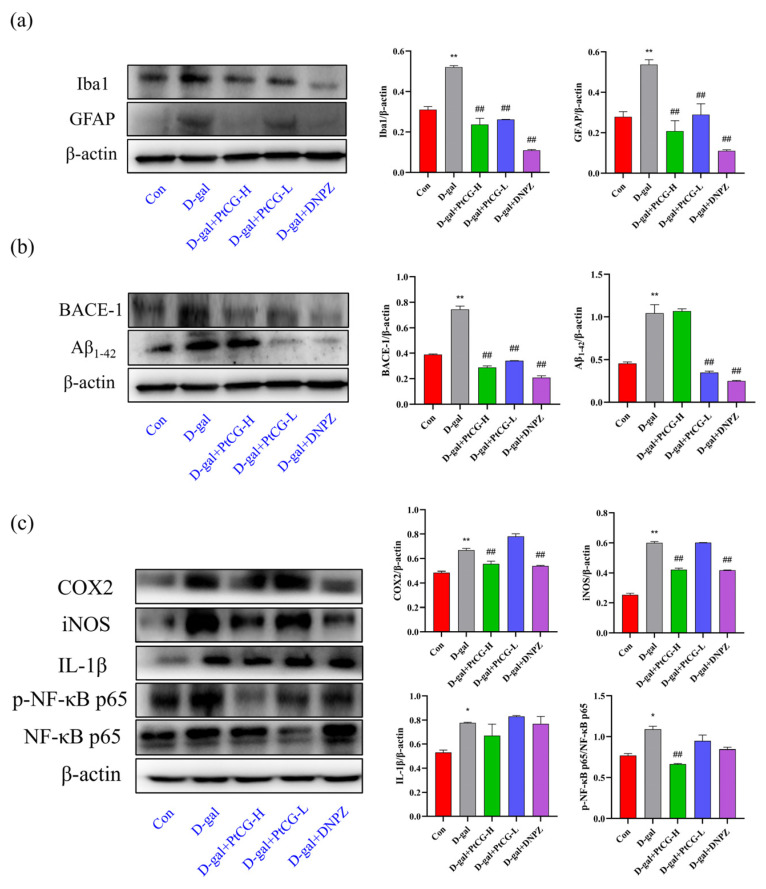
PtCG downregulated the expression of related proteins in the hippocampus of aging mice. (**a**) Protein expression levels of Iba1 and GFAP; (**b**) protein expression levels of BACE-1 and Aβ_1–42_; (**c**) protein expression levels of the NF-κB inflammatory signaling pathway. Data are expressed as mean ± SD (n = 3 per group). * *p* < 0.05 and ** *p* < 0.01 vs. Con group; ^##^ *p* < 0.01 vs. D-gal group.

According to recent research, microglia and astrocytes play a critical role in neuroinflammatory diseases. These two types of glial cells are the predominant nonneuronal cells in the central nervous system and not only maintain the normal function of the nervous system but also exert an important regulatory role in inflammatory responses and disease processes. GFAP can be used as a marker of astrocyte proliferation, while Iba-1 can be employed as a marker of microglia activation [28,29,30,31]. The levels of GFAP and Iba-1 protein expression were assessed to identify gliosis in the mouse hippocampal tissues. Western blot analysis revealed that the model group had significantly higher expression levels of GFAP and Iba-1 protein in the hippocampus compared to the control group (*p* < 0.01, F (4, 5) = 37.02, F (4, 5) = 188.0). PtCG administration significantly reduced the expression of GFAP and Iba-1 in a dose-dependent manner (*p* < 0.01), comparable to DNPZ. These results suggest that D-gal treatment stimulated microglia and astrocyte hyperplasia in the hippocampus of mice, while PtCG and DNPZ inhibited gliosis, thereby alleviating the damage induced by D-gal (Figure 6a).

### 2.7. PtCG Decreased the Protein Expression of Aβ_1–42_ and BACE-1 in the Hippocampus of Aging Mice

BACE-1 is the most crucial β- and γ-secretase in amyloid precursor protein (APP) processing. It is particularly abundant in various neuronal cell types, especially in response to injury and inflammation, playing a vital role in maintenance and repair. The abnormal cleavage of APP primarily produces Aβ, and Aβ deposition can exacerbate cellular senescence. Clinical trials have demonstrated that increased levels of BACE-1 lead to enhanced Aβ production [32,33,34,35,36]. Western blot analysis revealed a considerably higher expression of BACE-1 and Aβ_1–42_ in the hippocampus of the model group compared to the control group (*p* < 0.01, F (4, 5) = 386.8, F (4, 5) = 132.1). The expression of BACE-1 and Aβ protein was significantly downregulated in both the PtCG-L and DNPZ groups (Figure 6b) (*p* < 0.01).

### 2.8. PtCG Downregulated the Expression Inflammatory-Related Proteins in the Hippocampus of Aging Mice

NF-κB is linked to cancer, neurodegeneration, and inflammation. Prior studies have shown that D-gal can activate the NF-κB signaling pathway in the hippocampus and cortex of rats, resulting in inflammatory responses [37]. Western blot analysis was employed to examine the expression of inflammatory markers in the hippocampus, including COX-2, iNOS, IL-1β, and p65. D-gal injection significantly increased the expression of COX-2 (*p* < 0.01, F (4, 5) = 119.3), iNOS (*p* < 0.01, F (4, 5) = 716.0), IL-1β (*p* < 0.05, F (4, 5) = 10.26), and p65 (*p* < 0.05, *p* < 0.01, F (4, 5) = 35.56) in the hippocampus compared to the control group. The administration of PtCG-H and DNPZ significantly attenuated these increases in the hippocampus (*p* < 0.01). However, the protein levels in the hippocampus of the PtCG and DNPZ groups did not return to normal levels (Figure 6c).

## 3. Discussion

The natural aging model is one of the most effective models for studying brain aging. Researchers have also extensively utilized the accelerated aging paradigm in mice, which is induced by injecting substantial amounts of D-gal [25,38]. During the aging process, the functions of critical organs gradually decline due to the attack of reactive oxygen species (ROS) [39]. The brain, being the most complex tissue in the human body, requires a significant amount of energy and oxygen to function normally [40,41]. Therefore, it is widely accepted that administering D-gal to rats for 8 weeks can establish an aging model. Applying this model to study the effects of supplements and antioxidants could potentially lead to the development of novel therapies targeting age-related brain dysfunction.

Aβ is a small peptide produced by the hydrolysis of APP. BACE-1 is the key rate-limiting enzyme in the process of Aβ production [42,43]. APP was converted into insoluble Aβ, which accelerated the deposition of Aβ in the brain of pathological mice [44]. The substantial presence of microglia and astrocytes around Aβ plaques also suggests that Aβ deposition may be highly correlated with inflammatory responses. Neuroglial cells, particularly microglia and astrocytes, are independent of each other but closely linked, maintaining the homeostasis of the brain’s innate immune system and microenvironment. Studies have demonstrated that activated microglia and astrocytes play a crucial role in inducing neuroinflammatory response pathways [45,46,47]. The overactivation of microglia and astrocytes produces high levels of pro-inflammatory factors, leading to neuroinflammatory and neurotoxic responses [48]. In our study, the levels of Aβ and BACE-1 protein were significantly reduced by PtCG treatment, suggesting that PtCG might play an anti-aging role by inhibiting the BACE-1 and Aβ signaling pathways.

The NF-κB protein family is a group of significant transcription factors involved in inflammation, oxidative stress, and cell damage. This family is closely linked to various physiological processes in the organism. When stimulated by oxidative stress and inflammatory factors, NF-κB can further produce inflammatory factors, leading to cellular damage due to the body’s prolonged hyperinflammatory state. Some researchers propose that free radicals and inflammation are the primary causes of aging, with the NF-κB signaling pathway being the main inflammatory signaling pathway [49]. ROS are associated with cytokines such as IL-1β and facilitate the translocation of activated NF-κB to the nucleus, controlling the expression of pro-inflammatory proteins like iNOS and COX2. These factors can cause tissue and organ damage and are involved in apoptosis, inflammation, and immune responses [50,51]. In our study, the levels of these inflammatory factors were significantly lower in the PtCG-treated mice compared to the D-gal model group, suggesting that PtCG may possess anti-inflammatory properties in D-gal-stimulated mice.

Numerous studies have demonstrated that the long-term administration of D-gal can mimic the pathology of aging and induce liver and kidney damage [52]. D-gal-induced oxidative damage results in the increased generation of ROS, decreased levels of antioxidant enzymes, and inflammatory reactions in the liver and kidneys [53]. Due to their specialized roles in metabolism and detoxification, the liver and kidneys are particularly susceptible to D-gal toxicity, with the liver being the primary site of D-gal metabolism [40,41]. Exposure to D-gal elevates intracellular ROS production and lipid peroxidation in both liver and kidney tissues. ALT and AST are widely used clinical biomarkers for assessing hepatocellular injury [54]. Experimental results indicate that D-gal injection can cause liver function impairment, hepatocyte degeneration, and inflammatory cell infiltration, which can be alleviated by treatment with PtCG. PtCG treatment effectively increases the activities of AKP, AST, and ALT, and can partially restore histopathological changes in the liver. BUN is a key clinical indicator of renal function [55]. The experimental findings reveal that D-gal infusion can cause renal impairment, which can be significantly improved by continuous PtCG treatment, indicating that PtCG administration protects both the liver and kidneys from D-gal-induced damage in mice.

**Figure 7:**
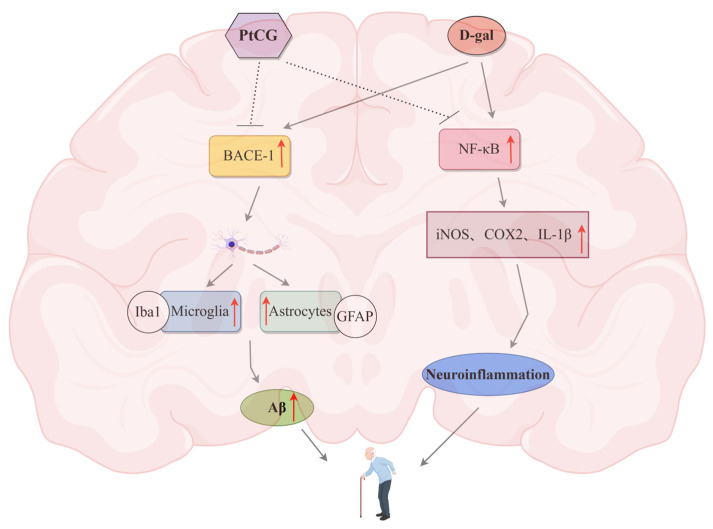
Schematic diagram of anti-aging mechanism of PtCG. Note: Red up arrows represent upregulation of expression. Grey dashed arrows represent the inhibitory effect. Solid arrows represent promoting effect. (The mechanism diagram was drawn by using Figdraw 2.0 (24 September 2024).)

Oxidative stress is a primary contributor to numerous diseases, with elevated intracellular oxidative stress prevalent in many neurodegenerative disorders. [56]. When injected into animals in large doses, D-gal is converted to galactitol, a compound that is difficult to further metabolize. The accumulation of galactitol in cells leads to changes in osmotic pressure, metabolic disruptions, increased intracellular ROS, and oxidative damage, which can precipitate various other major pathological changes and accelerate organismal aging [57]. The extent of oxidative damage can be estimated by assessing antioxidant enzyme activity and the levels of oxidative damage-associated products, such as MDA, a crucial marker of oxidative damage leading to lipid peroxidation (LPO) poisoning. As organisms age, antioxidant enzymes like SOD and GSH experience reduced functionality, diminishing their effectiveness in scavenging free radicals. This leads to an uncontrolled cascade of free radicals, ultimately increasing the final product of lipid peroxidation, MDA [58]. Our findings indicate that D-gal exposure results in elevated MDA levels in the serum and hippocampus of mice. However, even at a low dose of 50 mg/kg, PtCG attenuates oxidative damage. In summary, PtCG exerts neuroprotective effects, possibly by inhibiting the NF-κB signaling pathway and simultaneously reducing the expression of BACE-1, thereby inhibiting the proliferation of microglia and astrocytes and reducing the excessive accumulation of Aβ. These mechanisms contribute to the attenuation of D-gal-induced neurodegeneration, neuroinflammation, oxidative stress, and memory impairment (Figure 7).

## 4. Materials and Methods

### 4.1. Chemicals and Reagents

PtCG, the primary compound in *Lycium ruthenicum* Murr., was isolated according to a previously established method in our laboratory. Its purity was determined to be 97.38% by high-performance liquid chromatography (HPLC) at a wavelength of 520 nm [37]. D-galactose was obtained from Sigma-Aldrich (St. Louis, MO, USA). Donepezil was bought from Shanghai Macklin Biochemical Technology Co., Ltd. (Shanghai, China). The superoxide dismutase (SOD), glutathione (GSH), uric acid (UA), blood urea nitrogen (BUN), alanine aminotransferase (ALT), aspartate aminotransferase (AST), and alkaline phosphatase colorimetric assay kit (AKP) were all obtained from Nanjing Jiancheng Bioengineering Institute (Nanjing, China). Malondialdehyde (MDA), and bicinchoninic acid protein assay kits (BCA) were bought from Beyotime Biotechnology Co., Ltd. (Shanghai, China). AChE was obtained from Shanghai Enzyme-linked Biotechnology Co., Ltd. (Shanghai, China). Anti-Iba1, anti-GFAP, anti-β-actin, anti-BACE-1, and anti-Aβ_1–42_ were obtained from Abcam (Cambridge, MA, USA). Anti-NF-κB p65, anti-p-NF-κB p65, anti-COX2, anti-iNOS, and anti-IL-1β were obtained from Cell Signaling Technology (Danvers, MA, USA).

### 4.2. Animals and Experimental Design

SPF (specific-pathogen-free) grade female ICR mice (4-weeks-old, weight 18–22 g) were provided by SPF Biotechnology Co., Ltd. (Beijing, China) with the experimental animal production license number of SCXK (Jing) 2019-0010. The mice were housed in an IVC (individual ventilated caging) system with ad libitum access to water and food chow. The environment was maintained at a temperature of 23 ± 2 °C, a relative humidity of 55 ± 5%, and a 12-h light/dark cycle. The Northwest Plateau Institute of Biology, Chinese Academy of Sciences (CAS), approved all protocols employed in the animal research. 

**Figure 8:**
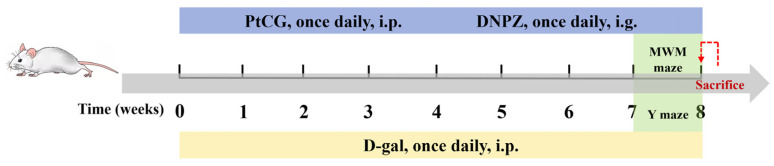
Experimental procedure and treatment schedule.

Following a one-week acclimatization period, the mice were randomly assigned to 6 groups: (n = 10): Control (Con), D-gal (100 mg/kg, ip), PtCG-H (100 mg/kg, ip), PtCG-L (50 mg/kg, ip), and DNPZ (5 mg/kg, ig). Over the course of eight weeks, the D-gal, PtCG, and DNPZ groups received a single daily treatment of their respective interventions (Figure 8).

### 4.3. Behavioral Tests

The behavioral experiments were conducted during the seventh week and continued for one week.

#### 4.3.1. Morris Water Maze Test (MWM)

The MWM test was employed to evaluate spatial learning and memory in mice [59]. The water maze apparatus consisted of a circular pool, a circular hidden escape platform, and a recording system. The circular escape platform, which served as the target quadrant of the pool, was located in the center of the third quadrant. The test was conducted in a dimly illuminated environment, with curtains surrounding the maze. The MWM test was performed daily between 9 a.m. and 5 p.m.

Prior to treatment, the mice underwent a five-day consecutive training regimen for orientation and navigation tests. Each day consisted of four consecutive training trials, with the mice’s entry point into the pool randomized for each trial. The mice were placed in the pool facing the wall, and the recording device was activated to measure the escape latency and track the swim route until the mice reached the platform. If a mouse failed to locate the platform within 120 s, it was guided to the platform and held there for 15 s. On the final day of trials, each mouse was allowed 120 s of unrestricted swimming in the pool without a platform. The number of times the mouse reached the target platform position, the duration spent in the target quadrant, and the swimming speed were recorded [26].

#### 4.3.2. Y-Maze Tests

The Y-maze test was employed to assess exploratory behavior and short-term spatial memory in mice. The Y-maze, a horizontal maze with three arms converging at equal angles, allowed each mouse to freely explore for an eight-minute period. The mice were given a consistent starting point at the end of one arm. To eliminate residual odors from previous trials, the arms were thoroughly cleaned with 75% ethanol between each trial. The test was conducted in a dimly illuminated environment, with curtains surrounding the maze. The specific time that the Y-maze tests were conducted was between 9 a.m. and 3 p.m. daily. Alternation was defined as consecutive entries into the three arms on overlapping triplet sets. The total number and sequence of arm entries were recorded [60]. The percentage of alternation, which represents the proportion of arm choices differing from the previous two selections, was calculated using the following equation:

% alternations = (order of arm selections)/(overall arm selections − 2) × 100

### 4.4. Serum and Tissue Preparation

Following the completion of behavioral tests, all mice were allowed to rest for one day and underwent a 12-h fasting period. Blood samples were collected via the orbital vein and centrifuged at 3000 r/min (25 °C) for 15 min to obtain serum. The mice were then euthanized by cervical dislocation. The livers, kidneys, and brains were harvested on ice and rinsed with cold saline. Hippocampal tissues were dissected from the brains. All serum and tissue samples were stored at −80 °C for further analysis. Liver tissues designated for H&E staining and Masson staining were preserved in 4% paraformaldehyde. Similarly, hippocampal tissues intended for H&E staining and Nissl staining were fixed in 4% paraformaldehyde. Hippocampal samples selected for TEM analysis were stored in a specialized fixative designed for electron microscopy.

### 4.5. Biochemical Analysis

In accordance with the manufacturer’s recommendations, the following parameters were measured: BUN and UA in the kidneys, AKP, AST, and ALT in the liver, and SOD, GSH, MDA, and AChE in the serum and hippocampus.

### 4.6. Pathological Analysis

#### 4.6.1. Transmission Electron Microscopy (TEM)

Hippocampal samples were rinsed, fixed, dehydrated, embedded, and stained according to established protocols in the literature [61,62]. Using TEM, researchers observed and captured images of morphological changes in synaptic vesicles, quantified the number of synapses, and measured the thickness of the PSD in the hippocampal samples (HT7800, HITACHI, Tokyo, Japan).

#### 4.6.2. H&E Staining

The livers and brains were fixed in 4% paraformaldehyde for 24 h before undergoing dehydration with gradient ethanol, embedding in paraffin, and sectioning. Following the cutting process, the tissue sections were immersed in warm water, mounted on glass slides, dewaxed, and stained with H&E. All stained tissue sections were examined under a light microscope to observe histopathological changes, followed by image acquisition and analysis [63].

#### 4.6.3. Nissl Staining

The brain sections were subjected to two rounds of xylene staining, each lasting 20 min, followed by a gradient ethanol dehydration process. Subsequently, the sections were stained with toluidine blue for a duration of 2–5 min, treated with a 1% solution of glacial acetic acid, and then dried in an oven. The sections were then exposed to xylene for 10 min before being sealed with neutral gum [64,65]. Finally, microscopic inspection was conducted to observe changes in the hippocampus, and image acquisition and analysis were performed to further examine the findings.

#### 4.6.4. Masson Staining

Liver sections were rinsed, immersed, infected, differentiated, dehydrated, and stained. The prepared pathological tissue slices were examined using a light microscope (Nikon Eclipse E100, Tokyo, Japan).

### 4.7. Western Blot Analysis

The hippocampal protein was extracted on ice using a lysis solution containing protease and phosphatase inhibitor cocktails. The mixture was centrifuged at 12,000× *g* for 15 min at 4 °C, and the resulting supernatant was collected. The BCA assay kit was utilized to identify and quantify the protein content in the supernatant [66].

After completely melting the hippocampus protein samples, a 5× loading buffer was added. The samples were denatured in a metal bath at 100 °C for 10 min. The total amount of protein sample used for SDS-PAGE electrophoresis was 25–30 μg. Subsequently, the protein was transferred to PVDF membranes using a membrane transfer device, and the membranes were blocked for 1.5 h at room temperature using TBS-T buffer containing 5% skim milk. The PVDF membranes containing the target bands were incubated at 4 °C overnight and then washed three times with TBS-T solution, with a 10-min interval between each wash. Following a 1-h incubation period at room temperature with the secondary antibody, the PVDF membranes were washed three times with TBS-T solution, each wash lasting 10 min. The targeted bands were detected using the ECL luminescence technique with the 5200 Multi Luminescent imaging systems (Tanon, Shanghai, China). The gray value of the protein was assessed using ImageJ-win64 (NIH, Bethesda, MD, USA).

### 4.8. Statistical Analysis

GraphPad Prism 8.0 (GraphPad Software, Inc., La Jolla, CA, USA), a statistical software package, was employed to conduct the analysis. Data were expressed as mean ± standard deviation (SD). Differences between groups were assessed using one-way analysis of variance (ANOVA) followed by Tukey’s post hoc test for multiple comparisons. ^#^ *p* < 0.05 and ^##^ *p* < 0.01 were compared with the Con group. ^∗^ *p* < 0.05 and ^∗∗^ *p* < 0.01 were compared with the D-gal group.

## 5. Conclusions

This experiment investigated the anti-aging effects of PtCG in a D-gal-induced aging mouse model. The results demonstrated that PtCG treatment ameliorated learning and memory deficits, mitigated neuronal damage by suppressing neuroinflammation and oxidative stress, alleviated liver and kidney injuries, inhibited glial cell proliferation, and reduced the excessive accumulation of Aβ and BACE-1. Notably, PtCG exhibited superior effects compared to the positive control drug, DNPZ. Collectively, these findings suggest that PtCG possesses potential therapeutic efficacy in attenuating aging-associated symptoms.

## Data Availability

The original contributions presented in the study are included in the article, further inquiries can be directed to the corresponding author.
